# Nanoarchitectonics of Nanocellulose Filament Electrodes by Femtosecond Pulse Laser Deposition of ZnO and *In Situ* Conjugation of Conductive Polymers

**DOI:** 10.1021/acsami.4c02780

**Published:** 2024-04-17

**Authors:** Duong Tuan Anh Nguyen, Ling Wang, Toyoko Imae, Chun-Jen Su, U-Ser Jeng, Orlando J. Rojas

**Affiliations:** † Graduate Institute of Applied Science and Technology, National Taiwan University of Science and Technology, Taipei 10607, Taiwan; ‡ Department of Bioproducts and Biosystems, School of Chemical Engineering, Aalto University, 00076 Espoo, Finland; § Department of Chemical Engineering, National Taiwan University of Science and Technology, Taipei 10607, Taiwan; ∥ National Synchrotron Radiation Research Center, Hsinchu 300092, Taiwan; ⊥ Department of Chemical Engineering, National Tsing Hua University, Hsinchu 300044, Taiwan; ^#^Department of Chemical and Biological Engineering, ^∇^Department of Chemistry, and ^○^Department of Wood Science, Bioproducts Institute, University of British Columbia, Vancouver, British Columbia V6T 1Z3, Canada

**Keywords:** conductive filaments, cellulose nanofibrils, electrodes, zinc oxide, conjugated polymer, synergistic performance

## Abstract

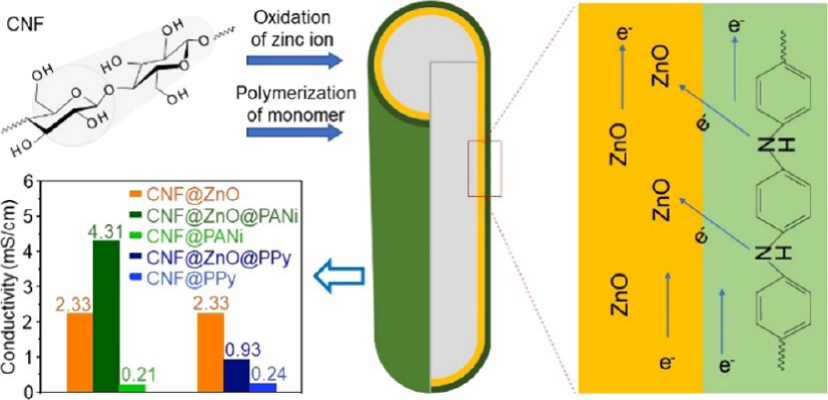

Electroactive filament electrodes were synthesized by wet-spinning of cellulose nanofibrils (CNF) followed by femtosecond pulse laser deposition of ZnO (CNF@ZnO). A layer of conducting conjugated polymers was further adsorbed by *in situ* polymerization of either pyrrole or aniline, yielding systems optimized for electron conduction. The resultant hybrid filaments were thoroughly characterized by imaging, spectroscopy, electrochemical impedance, and small- and wide-angle X-ray scattering. For the filaments using polyaniline, the measured conductivity was a result of the synergy between the inorganic and organic layers, while the contribution was additive in the case of the systems containing polypyrrole. This observation is rationalized by the occurrence of charge transfer between ZnO and polyaniline but not that with polypyrrole. The introduced conductive hybrid filaments displayed a performance that competes with that of metallic counterparts, offering great promise for next-generation filament electrodes based on renewable nanocellulose.

## 1 Introduction

In the past decade, bioresources have been considered as alternatives to mineral or petrochemical-derived counterparts to produce functional and energy materials. The recent developments in the area, particularly related to sustainable cellulose-based nanomaterials, have shown promise for their impact in improving the quality of life, which depends on advanced functional materials, environmental protection, energy security, and health.^1^ In this context, energy generation and storage technologies have played a critical role due to the rapid development of wearable and portable devices. Especially, cellulose nanofibrils (CNFs), which are widely available, lightweight, low cost, flexible, and biodegradable, have been integrated into energy storage devices.^2^ Thus, CNFs have been tested as support or templating material for supercapacitors and solar cell devices.^3,4^ Herein, we address a relevant and serious limitation as far as CNF properties as an insulator for adsorption of conducting polymers and metal oxides. This is a requirement in many advanced applications, for instance, in conducting yarns or textiles that benefit from the inherent properties of CNF, for instance, in photovoltaic and photocatalytic systems. We introduce a femtosecond pulse laser to couple the organic phase with ZnO particles that are not only conductive but also offer the possibility of developing other electroactive properties. Importantly, the femtosecond pulse laser synthesis proceeded by OH* evolution, where the photoenergy of the laser itself modulated the main reaction, leading to small particles compared to those obtained by hydrothermal alternatives. We also offer the possibility of polymer conjugation and demonstrate a remarkable performance due to the synergistic effects of the two conductive phases, opening new opportunities for CNF-based hybrid systems.

Among conductive polymers, polyaniline (PANi) is a highly conductive polymer with high specific capacitance from multiredox reactions. PANi displays excellent thermal stability and is easily synthesized.^5^ Polypyrrole (PPy), on the other hand, displays low toxicity, outstanding redox-active performance, and high conductivity.^6−8^ Finally, ZnO has received considerable interest as a superior semiconductor and is useful for its antibacterial and photocatalytic properties in dye-sensitized solar cells and sensors.^9−12^ Various techniques have been used to prepare ZnO, including mechanical methods, polyol deposition, sol–gel synthesis, thermal decomposition, spray pyrolysis, hydrothermal synthesis, and femtosecond pulse laser irradiation.^12−17^ The bottom-up synthesis by means of the femtosecond pulse laser irradiation has been demonstrated to yield small-sized clusters of ZnO in slightly acidic aqueous media with a quantum effect and a large surface area.^12^ The products from this route display large bandgap, fast charge transfer, strong photoluminescence, and effective photocatalytic activity compared to those produced under alkaline conditions and from the conventional hydrothermal synthesis.

Early reports have discussed the deposition of polyaniline on nanocellulose and natural rubber nanocomposites^18^ as well as on 3D-conductive CNF aerogels,^19^ indicating electrical conductivities suitable for semiconductor devices.^20,21^ In this work, ZnO was deposited on CNF filaments based on both femtosecond pulse laser irradiation and polyol deposition. Hybrid filaments were obtained as a single system integrating either ZnO or conductive polymers (PANi and PPy). Filaments with double layers were also produced by combining ZnO with one of the two conductive polymers. The conduction characteristics of different composition permutations (CNF@ZnO, CNF@PANi, CNF@PPy, CNF@ZnO@PANi, and CNF@ZnO@PPy) were compared. These new materials are found to have a remarkable performance by the synergistic effects of the two conductive phases, opening new opportunities for CNF-based hybrid systems with conductivity.

## 2 Experimental Section

### 2.1 Materials and Methods

Zinc nitrate hexahydrate (Zn(NO_3_)_2_·6H_2_O, 98%), ethylene glycol ((CH_2_OH)_2_, 99%), aniline monomer (99% extra pure), pyrrole monomer (99% extra pure), and ammonium persulfate (APS, 98%) were sourced from Acros Organics, Belgium. Other reagents were of commercial grade. Doubly distilled deionized water with a resistivity of 18 MΩ cm was obtained from a Yamato Millipore WT100, Japan.

A femtosecond laser (mode-locked Er fiber laser kit, Kokyo Inc., Japan) was operated by means of a laser diode (THORLABS BL976-SAG300) using a wavelength of 976 nm, minimum power of 300 mW, and typical current of 470 mA. A femtosecond pulse laser was created from the incident laser based on the mode-locking method and amplified using a chirped pulse amplifier. The system included a pulse laser amplifier operated at a 1550 nm wavelength (pulse width of several hundred femtoseconds and 45 MHz repeat frequency) with a constant power output of 47 mW. The FTIR absorption spectra were recorded with KBr pellets on a Thermo Scientific Nicolet 6700 spectrometer in a wavenumber range of 4000–450 cm^–1^ (resolution of 2 cm^–1^ and 64 scans). A field-emission scanning electron microscope (FE-SEM, JEOL, JSM6500F, Japan) equipped with energy dispersive X-ray (EDX) diffraction and X-ray energy dispersion mapping was operated at an accelerating voltage of 10 kV.

The electrochemical impedance spectra (EIS) were acquired by using a Zahner Zennium E electrochemical workstation (Germany) with 1 Hz–1 MHz frequencies and a 50-mV amplitude in a three-electrode system, where a working electrode was connected with a Ag/AgCl reference and platinum counter electrodes in 1 M NaCl. In the analysis, the conductivity (σ) value for the electrodes was taken as the inverse ratio of the charge transfer resistance (*R*_ct_), following the expression:^22^
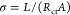
1where *L* is the electrode height in the electrolyte (1 cm). *A* is the total surface area of the cylindrical filament electrode in contact with the electrolyte, including the lateral and bottom surface area, *A*, calculated by the following expression:
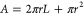
2with the radius (*r*) of the filament electrode determined by FE-SEM imaging.

Small-angle and wide-angle X-ray scattering (SAXS and WAXS) experiments were carried out using the High-Energy Wiggler Beamline 23 A of the Taiwan Light Source in the National Synchrotron Radiation Research Center, Hsinchu, Taiwan.^23^ The SAXS and WAXS data were collected using a Pilatus 1M-F detector of 172 × 172 μm^2^ pixel resolution (Dectris Co., Switzerland) and a flat panel detector of 50 × 50 μm^2^ pixel resolution (Hamamatsu C9728DK, Japan), respectively.

### 2.2 Filament Synthesis

CNF filaments were prepared according to an earlier report.^24^ In a typical procedure, never-dried bleached birch fibers (UPM Pietarsaari Mill, Finland) were oxidized at pH 10 with 2,2,6,6-tetramethylpiperidine-1-oxyl (TEMPO, Sigma-Aldrich, Germany) and microfluidized (Microfluidics Corp.) at a high pressure (2000 bar). The TEMPO-oxidized cellulose nanofibrils (TOCNF, for simplicity, herein referred to as CNF) were used to produce filaments in a wet-spinning system.^25^ In detail, a CNF hydrogel was placed in a syringe and pumped through a tubing (6 mm inner diameter) connected to a needle (1.2 × 40 mm^2^) and directly extruded into aqueous 1 M HCl solution. The spinning rate was set at 2 mL/min. The CNF wet filaments were collected on a rotating drum (22 cm diameter, 3.6 m/min) that also acted as a drier. The obtained filaments were washed following immersion in water and dried overnight in an oven at 60 °C. Then, CNF filaments were collected (average diameter of 100 μm).

#### 2.2.1 CNF@ZnO Filaments

Femtosecond pulse laser irradiation was used to deposit ZnO on the CNF filaments.^12^ For this purpose, the filaments were immersed in aqueous zinc nitrate hexahydrate solutions at given concentrations (0.01, 0.1, and 1 M). An aqueous 1 M NaOH solution was added dropwise into the suspension and kept overnight at room temperature. Then, the suspension with the filament was placed in a 10 mm path length quartz cell, irradiated with a femtosecond pulse laser for 2 min, and kept for different periods (holding times), namely, 1, 10, and 20 h. Under these conditions, protector-free ZnO nanoparticles (10–13 nm in size) were synthesized.^12^ The produced hybrid filaments (CNF@ZnO filaments) were rinsed with water and dried for 1 h at 60 °C.

An alternative polyol deposition method^26^ was also used: CNF filaments were immersed in ethylene glycol containing 1 M zinc nitrate hexahydrate during given adsorption periods (1, 10, and 20 h) at room temperature. An ethylene glycol solution with 2 M NaOH was added dropwise into the aforementioned suspension, and the mixture was stirred at 80 °C for different holding times (1, 5, 10, and 20 h). Ethylene glycol-protected ZnO nanoparticles (18 nm) were obtained. Finally, the suspension was cooled to room temperature without stirring, and the composite filament was then rinsed with water and dried for 1 h at 60 °C.

#### 2.2.2 CNF@PANi and CNF@PPy Filaments

For the preparation of CNF filaments coated with a conductive polymer, APS was dissolved in 1 M HCl (1 mL) at a mol ratio 1:1 of aniline to APS, and then the APS solution was added dropwise to the CNF@aniline dispersion cooled to 1–5 °C and kept for 5 h at the same temperature.^8^ Likewise, CNF@PANi filaments were prepared from the mixture, rinsed three times with water, and dried for 1 h at 60 °C. The resulting hybrid CNF@PANi filaments were prepared for different monomer volumes (10–150 μL using CNF, 5 mg). The same procedure was used to synthesize the CNF@PPy filaments.

#### 2.2.3 CNF@ZnO@PANi and CNF@ZnO@PPy Filaments

**Figure 1:**
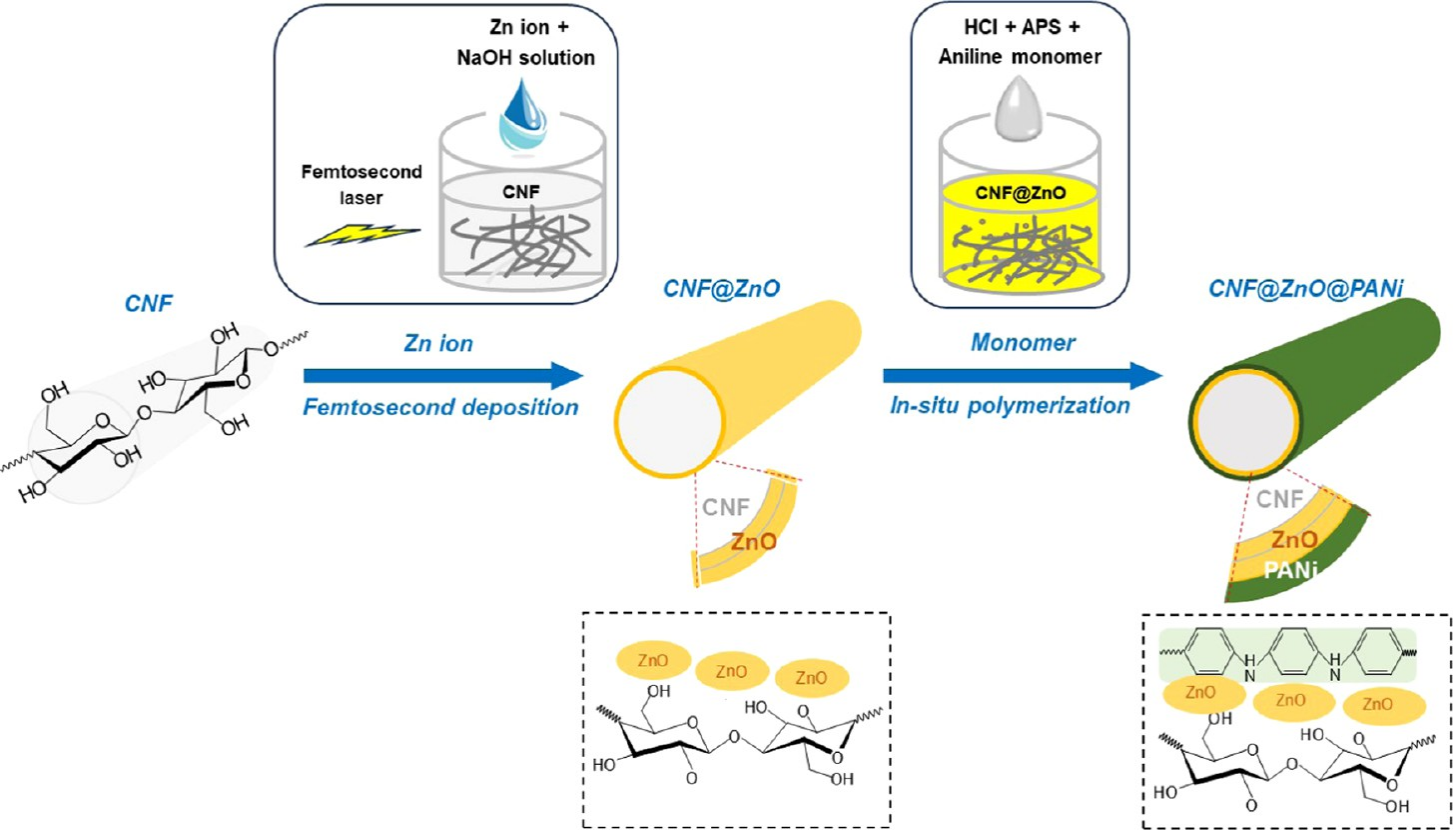
Schematic illustration of the preparation of the CNF@ZnO@PANi hybrid filament.

The preparation of CNF@ZnO@PANi and CNF@ZnO@PPy filaments was carried out with CNF@ZnO filaments prepared in a 0.1 M zinc nitrate hexahydrate solution and kept for 10 h after femtosecond laser irradiation. The aniline monomer was polymerized *in situ* by keeping the filament in 1 mL of aniline solution (including 75 μL of aniline monomer and 1 M HCl) for 1 h followed by 5-h treatment at 1–5 °C after the addition of 1 mL of solution of APS in HCl (at a mole ratio of 1:1 of aniline to APS). The CNF@ZnO@PANi hybrid filaments were collected from the mixture, rinsed three times with water, and dried for 1 h at 60 °C. The same experimental approach was used to create CNF@ZnO@PPy filaments using 100 μL of pyrrole monomer. Figure 1 includes a schematic illustration followed by the synthesis of CNF@ZnO@PANi.

## 3 Results and Discussion

### 3.1 CNF@ZnO Filaments

#### 3.1.1 Femtosecond Laser Deposition of ZnO

**Figure 2:**
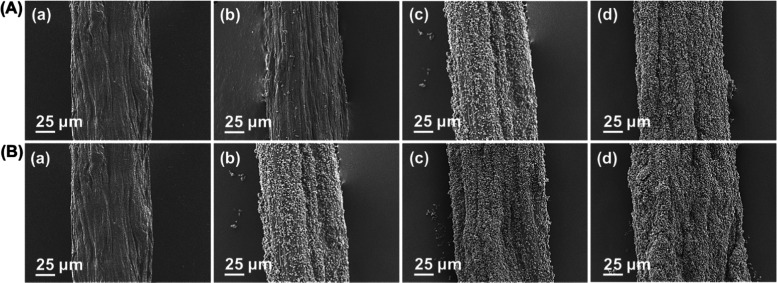
FE-SEM images of CNF@ZnO filaments prepared by femtosecond laser irradiation deposition at (A) different precursor concentrations: (a) 0 M, (b) 0.01 M, (c) 0.1 M, and (d) 1 M by using a fixed 1-h holding time. The results following different holding times after femtosecond laser irradiation are shown in panel (B): (a) 0 h, (b) 1 h, (c) 10 h, and (d) 20 h using a fixed 0.1 M precursor concentration.

The femtosecond laser deposition of ZnO on the CNF filaments was achieved by using the given Zn ion precursor at concentrations of 0.01, 0.1, and 1 M in 1 M NaOH (1-h holding period). FE-SEM images of CNF@ZnO filaments indicated that the deposition of ZnO on the filament was greatly affected by Zn ion concentration (Figure 2A): while the average diameter of the pristine CNF filament was ∼100 μm, a very thin ZnO layer was formed on the filament (∼102 μm thickness) at low precursor concentration (0.01 M). At increased concentrations, larger amounts of ZnO were anchored on the filaments: The respective CNF@ZnO filaments were ca. 109 and 117 μm in diameter for 0.1 and 1 M precursor concentrations, respectively. The effect of the holding period on ZnO deposition (from 0.1 M Zn ion concentration) after femtosecond pulse laser irradiation was also investigated (Figure 2B). The thickness of the CNF filament coated with ZnO increased to around 109, 124, and 142 μm for holding periods of 1, 10, and 20 h, respectively.

**Figure 3:**
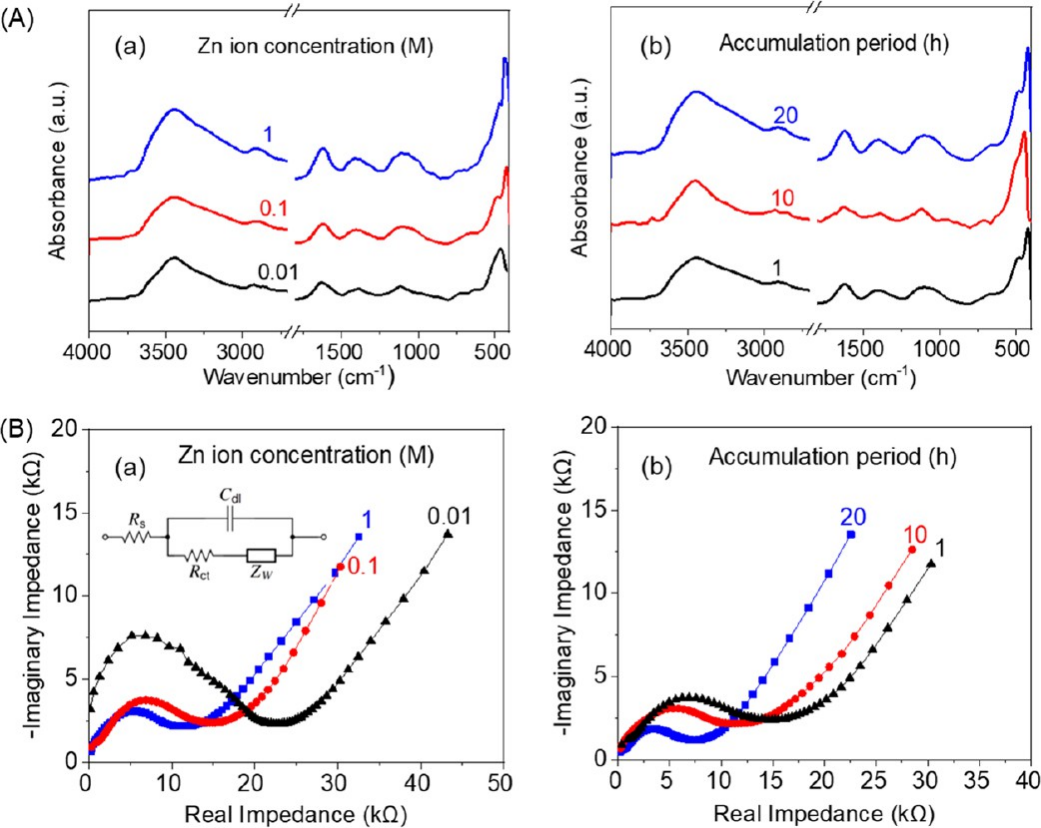
(A) FTIR absorption spectra and (B) EIS Nyquist plots of CNF@ZnO filaments prepared by femtosecond pulse laser irradiation at different (a) precursor concentrations (0.01, 0.1, and 1 M) and for a fixed 1-h holding time. (b) Results for different holding times (1, 10, and 20 h) after irradiation and 0.1 M precursor concentration are also shown.

The chemical characteristics of the obtained CNF@ZnO filaments were investigated using FTIR absorption spectrometry at different precursor concentrations and at different holding times after femtosecond laser irradiation. The spectra (Figure 3A) revealed the presence of the characteristic absorption bands of both CNF and ZnO. Specifically, characteristic absorption bands of O–H and C–H stretching vibration modes were observed at 3400 and 2899 cm^–1^, respectively. The COO^–^ stretching and O–H bending modes are attributed to the band at 1611 cm^–1^, and a band for C–H and C–O vibration modes in polysaccharide rings was located at ca. 1380 cm^–1^ along with a band at ca. 1100 cm^–1^ assigned to C–O–C out-of-plane bending. The Zn–O stretching mode of the ZnO layer on the CNF filament was assigned to the characteristic band appearing at 480 cm^–1^.^27,28^ In sum, the FTIR absorption spectra confirmed the coexistence of ZnO in the composite filaments, but no significant differences were observed for the conditions used. The existence of ZnO in the CNF@ZnO filament was further confirmed by EDX analyses (Figure S1), and elemental analysis indicated the existence of Zn, O, and C.

EIS data were collected to study the charge transfer performance of the filaments used as electrodes. Nyquist plots (Figure 3B) of CNF@ZnO filament electrodes showed semicircles in the high-frequency zone and a diffusion line in the low-frequency zone. The radius of the semicircle correlates with the magnitude of charge transfer resistance and thus can be used to assess the variation in charge transfer performance. The respective charge transfer resistance was obtained by fitting data to a Randles equivalent circuit model (inset of Figure 3B(a)) described by the charge transfer resistance (*R*_ct_), electrolyte resistance (*R*_s_), Warburg impedance (*Z*_W_), and double-layer capacitance (*C*_dl_).^29^ The conductivity was calculated (Table 1), and the CNF@ZnO filament electrodes prepared at different Zn ion concentrations (0.01, 0.1, and 1 M, respectively) indicated charge transfer resistances of 23.0, 13.5, and 10.5 kΩ, corresponding to conductivities of 1.35, 2.16, and 2.58 mS/cm. The holding time after femtosecond laser irradiation affected the charge transfer resistance: 13.5 and 7 kΩ for 1 and 20 h, respectively, corresponding to a conductivity of 2.16 and 3.19 mS/cm. These results indicated that the conductivity increased with the ZnO loading and holding time after irradiation.

**Table 1:** Parameters from EIS Curves of Filament Electrodes (CNF@ZnO, CNF @PANi, CNF @PPy, CNF@ZnO@PANi, and CNF@ZnO@PPy) Prepared Following the Given Methods and Conditions

filament and method		condition		thickness, μm	charge transfer resistance, kΩ	conductivity, mS/cm
CNF@ZnO	femtosecond laser irradiation method	Zn ion concentration, M	0.01	102	23	1.35
			0.1	109	13.5	2.16
			1	117	10.5	2.58
		holding time, h	1	109	13.5	2.16
			10	124	11.0	2.33
			20	142	7.0	3.19
	polyol method	adsorption time, h	1	107	635	0.05
			10	112	354	0.08
			20	119	244	0.11
		incubation time, h	1	119	244	0.11
			5	123	149	0.17
			10	129	62	0.40
			20	133	46	0.52
CNF@PANi			PANi 75 μL	123	121	0.21
CNF@PPy			PPy 100 μL	132	99	0.24
CNF@ZnO@PANi			PANi 75 μL	134	5.5	4.31
CNF@ZnO@PPy			PPy 100 μL	134	25.6	0.93

#### 3.1.2 ZnO Deposited in a Polyol Solvent

CNF@ZnO filaments were synthesized in an ethylene glycol solvent. FE-SEM images, IR absorption spectra, and EIS plots of CNF@ZnO filaments prepared at different precursor adsorption times (0, 1, 10, and 20 h) and given incubation times (1, 5, 10, and 20 h) are shown in Figures S2, S3 and S4, respectively. The obtained numerical values are summarized in Table 1 and indicate that the thickness of the hybrid filaments increased to 119 μm after 20 h of precursor adsorption. Further growth in thickness is seen for CNF@ZnO, reaching 133 μm at 20 h incubation. This value was lower than the largest filament thickness (142 μm) observed under the femtosecond pulse laser. EDX analyses confirmed the presence of Zn, O, and N elements. Since all of the IR absorption bands of CNF@ZnO filaments maintained the same wavelength, the IR spectra proved the presence of ZnO and CNF, although slight intensity variations occurred for the adsorption and incubation times used in the synthesis (Figure S4A). The values of the charge transfer resistance are calculated from the EIS Nyquist plots (Figure S4B) for CNF@ZnO filament electrodes prepared in the polyol solvent, which clearly decreased with the increased Zn ion precursor adsorption time on the CNF filaments and with the increased incubation time (Table 1). The conductivity increased with the reduced charge transfer resistance and reached a maximum value of 0.52 mS/cm at a 20-h incubation time. From Table 1, it can be concluded that between the two factors affecting the conductivity (eq 1), the reduction of charge transfer resistance dominates over the negative contribution of the increased thickness.

The mechanism for the formation of ZnO particles by femtosecond pulse laser irradiation is based on the production of hydroxyl radical (OH^•^), solvated electron (e_aq_^–^), and hydrogen radical (H^•^) from water molecules, which are exposed to the femtosecond pulse laser light.^30^ The contribution of hydroxyl radical to the formation of ZnO is confirmed using a scavenger,^12^ following the reaction of Zn^2+^ + 2OH^•^ → Zn(OH)_2_ → ZnO + H_2_O. The synthesized ZnO depends on the condition of the precursor solution: ZnO clusters were formed in the aqueous precursor solution, but nanoparticles resulted under alkaline conditions, noting that both clusters and nanoparticles are free from protector/stabilizers.^12^

In the synthesis of ZnO following the polyol method, Zn ions anchor on the adsorption sites (carboxylic groups of CNF) through electrostatic interactions and lone pair electrons in oxygen atoms of the carboxylic groups of the filament bind with Zn ions by electron pair sharing.^31,32^ When NaOH is added and the system is heated to 80 °C, the formation of ZnO particles on the filaments occurs by means of (1) hydrolysis of the preanchored Zn ions, generation of Zn(OH)_2_ in the presence of OH^–^ from NaOH;^11^ (2) condensation of hydrolyzed species, yielding a Zn–O–Zn bond by the removal of water upon heating; and (3) particle growth^33^ and size control by solvation of ethylene glycol used as a protector/stabilizer.

The CNF@ZnO filaments prepared by the two different procedures showed somewhat similar thicknesses. However, the conductivity was ca. 6-fold higher for filaments subjected to femtosecond pulse laser deposition than that processed with the polyol solvent. Possible reasons for these differences may relate to the synthesis procedure described above. The ZnO nanoparticles deposited by the femtosecond pulse laser were protector-free and 10–13 nm in size, and those synthesized in the polyol solvent were stabilized by ethylene glycol and were larger in size (18 nm).^12^ Smaller particles densely deposited on the CNF filaments transferred electrons fast. In addition, the protector-free semiconductors exhibited easier electron transfer, given the avoidance of disturbances by protector/stabilizers. The results indicate that ZnO deposition by femtosecond pulse laser irradiation is a preferable route. Therefore, CNF@ZnO filaments prepared by femtosecond pulse laser irradiation were considered in the following studies.

### 3.2 CNF@PANi and CNF@PPy Filaments

#### 3.2.1 CNF@PANi

**Figure 4:**
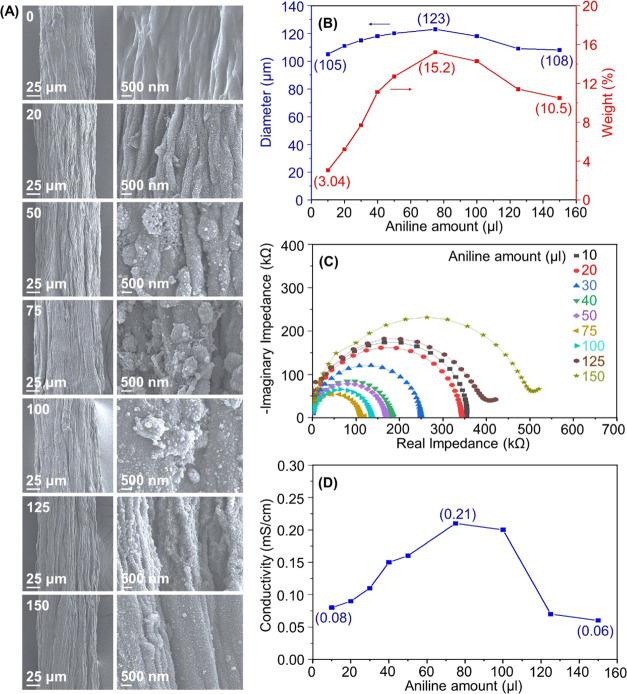
(A) FE-SEM and its higher magnified images, (B) diameter and PANi weight % changes, (C) Nyquist plots, and (D) conductivity of CNF@PANi filaments prepared by using different amounts of the aniline monomer (0–150 μL).

A smooth surface was observed for the initial *in situ* polymerization of aniline onto the CNF filament, suggesting that aniline was uniformly polymerized (Figure 4A). With increased aniline loading, the filament thickness increased (123 μm), and the filament surface was coated by particle deposition. However, the filament thickness gradually decreased and the particulate layer reduced when the amount of aniline exceeded 75 μL; that is, aniline was optimally deposited, leading to 123-μm CNF filaments. The weight variation of the PANi layer on the CNF filament was determined from the weight change of filaments before and after PANi deposition. The PANi content on CNF filaments gradually increased (Figure 4B): the increase in the amounts of the aniline monomer, by using a volume of 10 to 75 μL, led to a loading of 3.04–15.2 wt %, respectively. However, the loading was maximized at 75 μL, in agreement with the diameter variation of CNF@PANi filaments.

The impedance characteristics of CNF@PANi filaments were analyzed via the EIS Nyquist plots of electrodes prepared from different amounts of the aniline monomer (Figure 4C). The variation of semicircles in the Nyquist plots implied that the charge transfer resistance and thus the conductivity of filament electrodes correlated with the aniline loading. The calculated conductivity of CNF@PANi filaments increased from 0.09 to 0.21 mS/cm at 10–75 μL aniline solution volume (Figure 4D), corresponding to the charge transfer resistance from 358 to 121 kΩ. However, the conductivity tended to decrease when the amount of aniline exceeded 75 μL: It dropped from 0.21 mS/cm at 75 μL to 0.06 mS/cm at 150 μL. These changes can be attributed to the variation in thickness and PANi weight in the hybrid filaments.

#### 3.2.2 CNF@PPy

The pyrrole monomer was also deposited on the CNF filaments and polymerized *in situ* at different pyrrole amounts, forming PPy layers on the CNF filaments. The uniformity was remarkable for the CNF@PPy filaments at a low pyrrole amount (20 μL), but their surface became rough at medium and high pyrrole contents (75 and 100 μL, Figure S5A). The diameter of the hybrid filaments increased up to 132 μm, depending on the pyrrole amount, but it became thinner at higher pyrrole amounts and the roughness also decreased (Figure S5B). In the same way, the mass of PPy on the CNF filaments increased with the pyrrole amount and reached maximum (19.2 wt %) at 100 μL of the pyrrole monomer. The conductivity, which was evaluated from the semicircle in the EIS Nyquist plots (Figure S5C) of CNF@PPy filament electrodes at various amounts of the pyrrole monomer, similarly tracked with filament diameter and PPy mass, namely, the electrical conductivity was maximum (0.24 mS/cm) at 100 μL of the pyrrole monomer (Figure S5D), corresponding to a 99 kΩ charge transfer resistance. The results indicate that the filament diameter and the PPy weight were greatest at 100 μL of the pyrrole monomer and remarkably related to the conductivity of the filaments.

A shared property for CNF@PANi and CNF@PPy filaments is the observation of a maximum filament diameter at the intermediary polymer deposition. The deposited polymer weight and filament conductivity were maximized at the respective monomer loading, 75 μL and 100 μL, respectively (see Figures 4 and S5). The variation of conductivity depends on the competitive contributions of the filament radius and charge transfer resistance (eq 1). The numerical values of filament thickness, charge transfer resistance, and conductivity of CNF@PANi and CNF@PPy filaments are listed in Table S1. From Table 1, it can be concluded that the variation of conductivity is controlled by the variation of resistance but not the filament thickness. Then, the changes in conductivity relate to the resistance. The filament thickness tracks with the conductive polymer (PANi, PPy) loading in the insulative CNF. That is, the reduced thickness for conditions above the 75 μL PANi or 100 μL PPy owes to the lower conductive polymer loading, leading to increased resistance.

**Figure 5:**
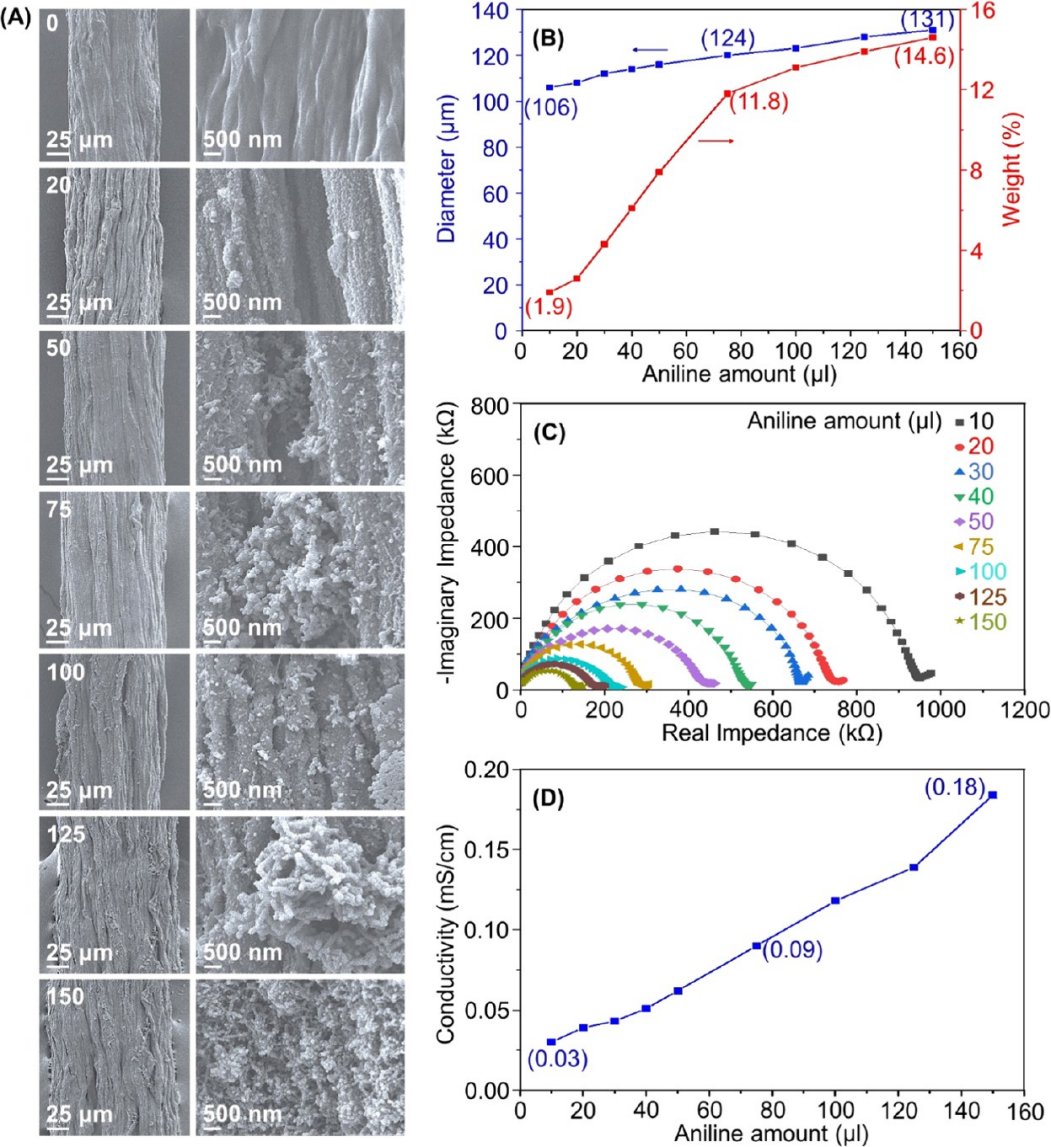
(A) FE-SEM and its magnified images, (B) diameter and weight % variations, (C) Nyquist plots, and (D) conductivity variations of CNF@PANi filaments prepared at different loadings of the aniline monomer (0–150 μL). APS was adsorbed on CNF before the addition of the monomer.

The observation of a maximum conductive polymer loading can be explained by the polymerization process through oxidation by APS, which occurs on the surface of the adsorbed monomer layer, leading to polymers that were anchored on the CNF filament. Assuming that the monomers were densely adsorbed on the CNF filament, at given optimum conditions, the polymerized terminal cannot reach the CNF filament for anchoring. Hence, nonanchored polymers may be removed from the CNF filament. It can be predicted that if a sufficient amount of APS was adsorbed on the CNF filament before the adsorption of the aniline monomer, then the polymerization on the CNF filament continuously thickens the PANi layer during polymerization. Figure 5 and Table S1 show the results of experiments to prove this point: Filament diameter, surface roughness, PANi weight, and filament conductivity increased with the increased aniline content. The respective values at 75 μL of the aniline content were 124 μm, 11.8 wt %, and 0.09 mS/cm, indicating similar thickness but slightly lower weight and conductivity values than those in Figure 4. The results suggest similar weight and conductivity values with twice the amount of the aniline monomer in the procedure used for CNF@PANi, according to Figure 5, compared with that corresponding to Figure 4.

**Figure 6:**
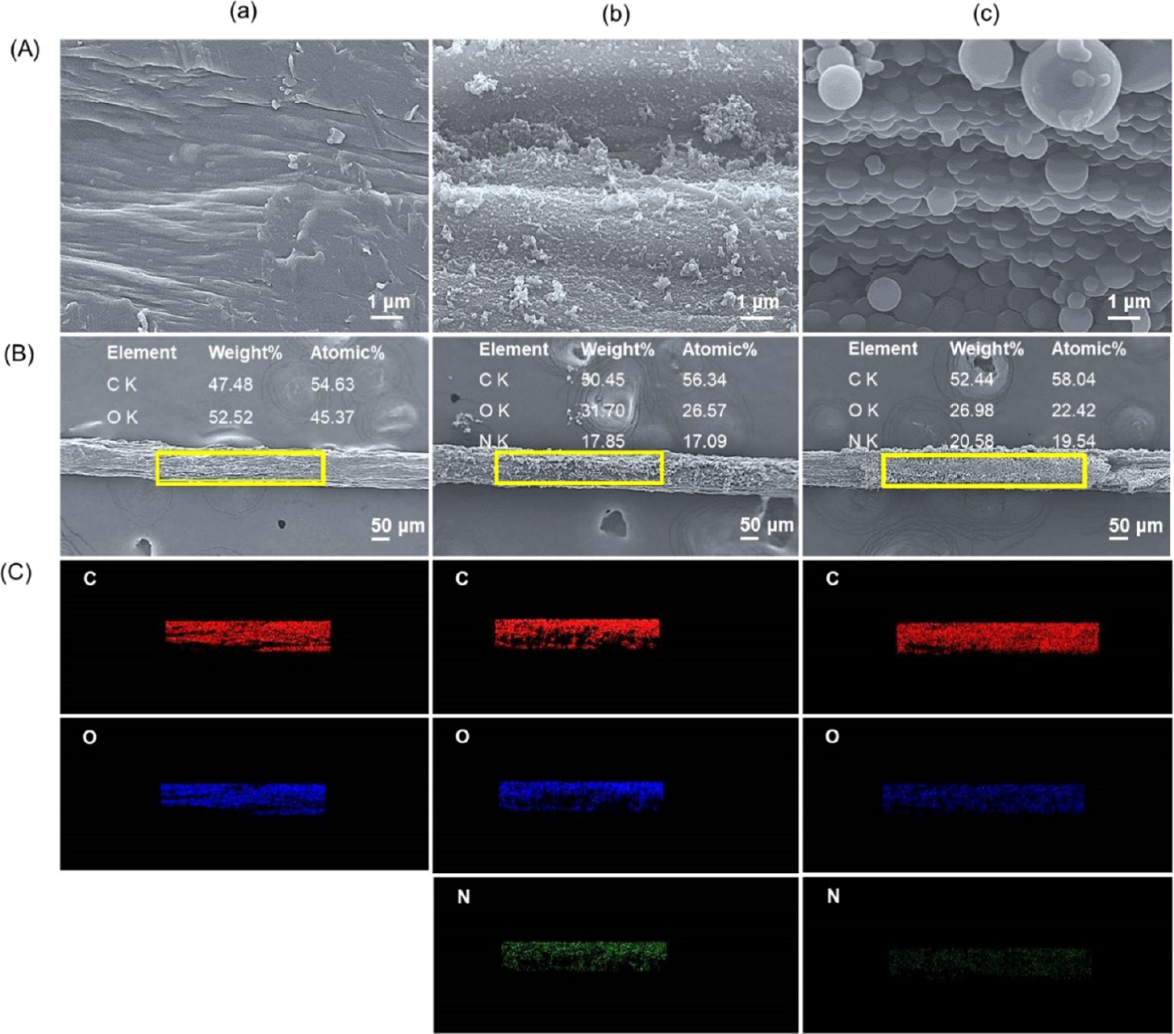
(A) FE-SEM images of the filament surfaces. (B) SEM images, elemental analyses, and (C) mapping images of carbon, oxygen, and nitrogen elements of (a) CNF, (b) CNF@PANi75, and (c) CNF@PPy100 filaments.

The morphological characteristics of CNF@PANi and CNF@PPy filaments against CNF were examined using FE-SEM. As seen in Figure 6A, the surfaces of CNF@PANi and CNF@PPy filaments were rougher than those of CNF filaments (PANi and PPy unevenly deposited on the CNF filaments). Elemental analysis (Figure 6B) showed that the atom % of carbon and oxygen in CNF was 54.6 and 45.4%, respectively, consistent with the chemical structures of CNF (see Figure 1). However, this ratio changed in CNF@PANi and CNF@PPy since PANi and PPy include carbon and nitrogen. In more detail, the elemental contributions of oxygen and nitrogen, respectively, were 26.6 and 17.09 atom % for PANi and 22.4 and 19.5 atom % for PPy. Since a unit of CNF and PANi or PPy is constructed by 10 oxygen and 1 nitrogen, respectively, the unit ratios of PANi/CNF and PPy/CNF are 17.09 × 10/26.57 = 6.4 and 19.54 × 10/22.42 = 8.7, indicating that the hybrid filaments were coated by a large amount of conducting polymers. The mappings shown in Figure 6C reflect the existence of carbon, oxygen, and nitrogen, demonstrating that the conductive polymers were successfully deposited on the CNF filaments.

**Figure 7:**
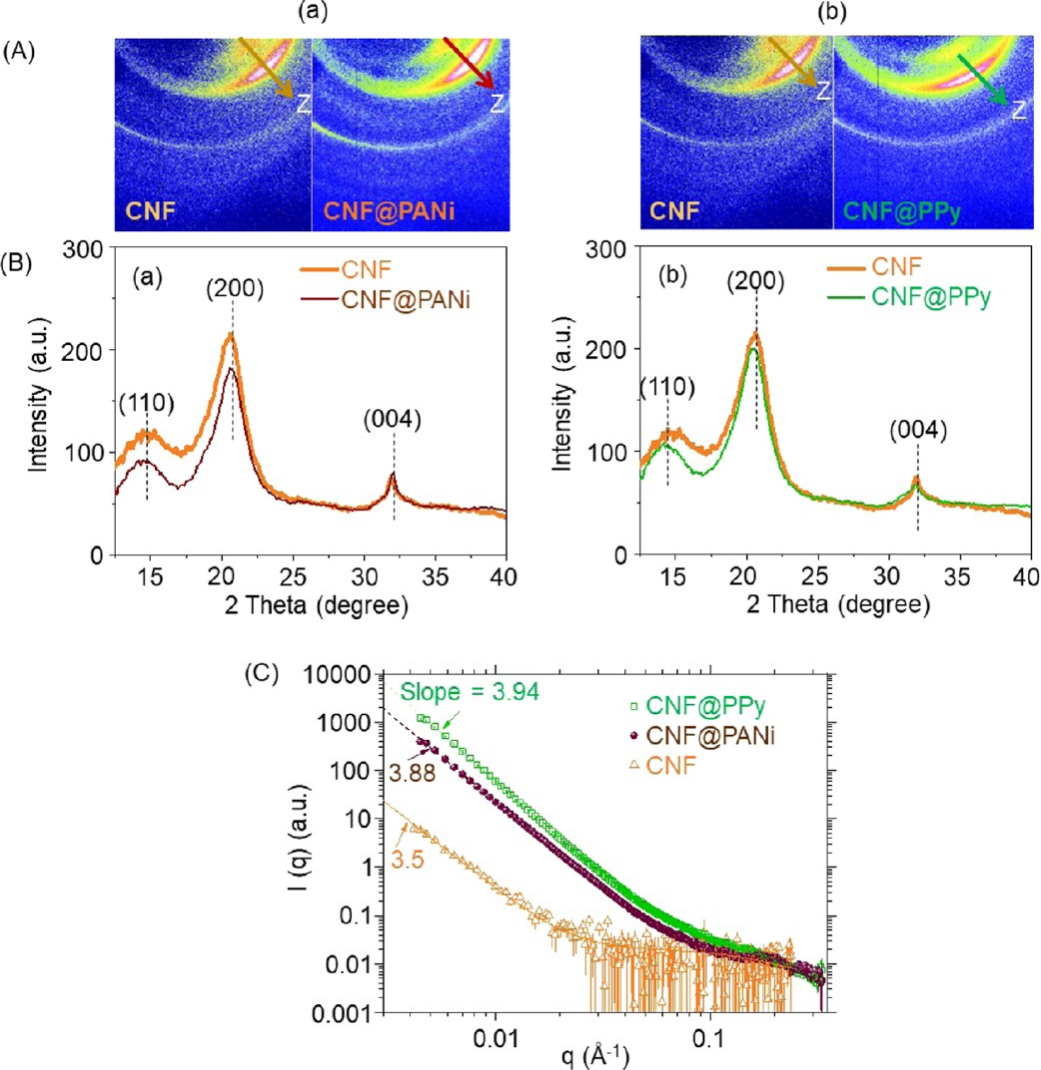
(A) WAXS patterns and (B) WAXS profiles showing highly oriented structures. A(a) and B(a) correspond to CNF@PANi, while A(b) and B(b) correspond to CNF@PPy (in both cases, CNF data is added for comparison). Arrows in panel (A) indicate the filament axis (*z*). Note that (110), (200), and (004) peaks result from the main contribution of the reflection planes of CNF. (C) SAXS profiles corresponding to CNF, CNF@PANi, and CNF@PPy fitted by power-low scattering (power indices as indicated).

SAXS/WAXS measurements were used to reveal the ordered structures in the CNF filaments and those after the two given treatments. Figure 7A(a) displays WAXS arcs of CNF and CNF@PANi filaments prepared with 10 μL aniline. The corresponding WAXS profiles shown in Figure 7B(a) exhibit the three characteristic peaks located at 2θ = 14.8, 21.2, and 32.1°, which are assigned to the (110), (200), and (004) reflection planes of CNF, respectively.^34,35^ The WAXS peak profile of the CNF@PANi overlapped with that of CNF, suggesting that the crystal packing structure of CNF was not significantly altered by PANi deposition, which has an amorphous structure, with no observable crystalline peaks.^36^ The amorphous PANi coating nevertheless contributed to an enhanced conductivity. Figure 7A(b),B(b) correspond to WAXS arcs and profiles of CNF and CNF@PPy filaments. Like in the case of PANi, the WAXS profiles of CNF@PPy overlapped with that of CNF, indicating the amorphous nature of the PPy overlayer.^37,38^

The SAXS profiles of CNF, CNF@PANi, and CNF@PPy filaments shown in Figure 7C feature a power-law scattering *I*(*q*) = *q*^–*n*^, in the low-*q* region, with fitted *n* values of 3.50, 3.88, and 3.94, respectively. These *n* values correspond to surface fractal dimensions, *D*_s_ = 6 – *n*, of 2.50, 2.12, and 2.06, respectively. The results suggest that the coating of PANi and PPy reduced significantly the large surface fractal dimension (*D*_s_ = 2.5) of CNF to a smaller dimension (*D*_s_ = 2.0).^39,40^ The surface fractal scattering features with large *n* values close to 4.0 or *D*_s_ close to 2.0 for the thick coating layers of PANi and PPy on CNF suggest that these layers were significantly smoother than that of CNF alone. We note that according to a previous report, very small and thin fibrils (few nanometers in cross-section) can form aggregates and mass fractal structures that follow a power-law scattering with *n* = 2.0 in the low-*q* region of the SAXS profile.^41^

### 3.3 CNF@ZnO@PANi and CNF@ZnO@PPy Filaments

**Figure 8:**
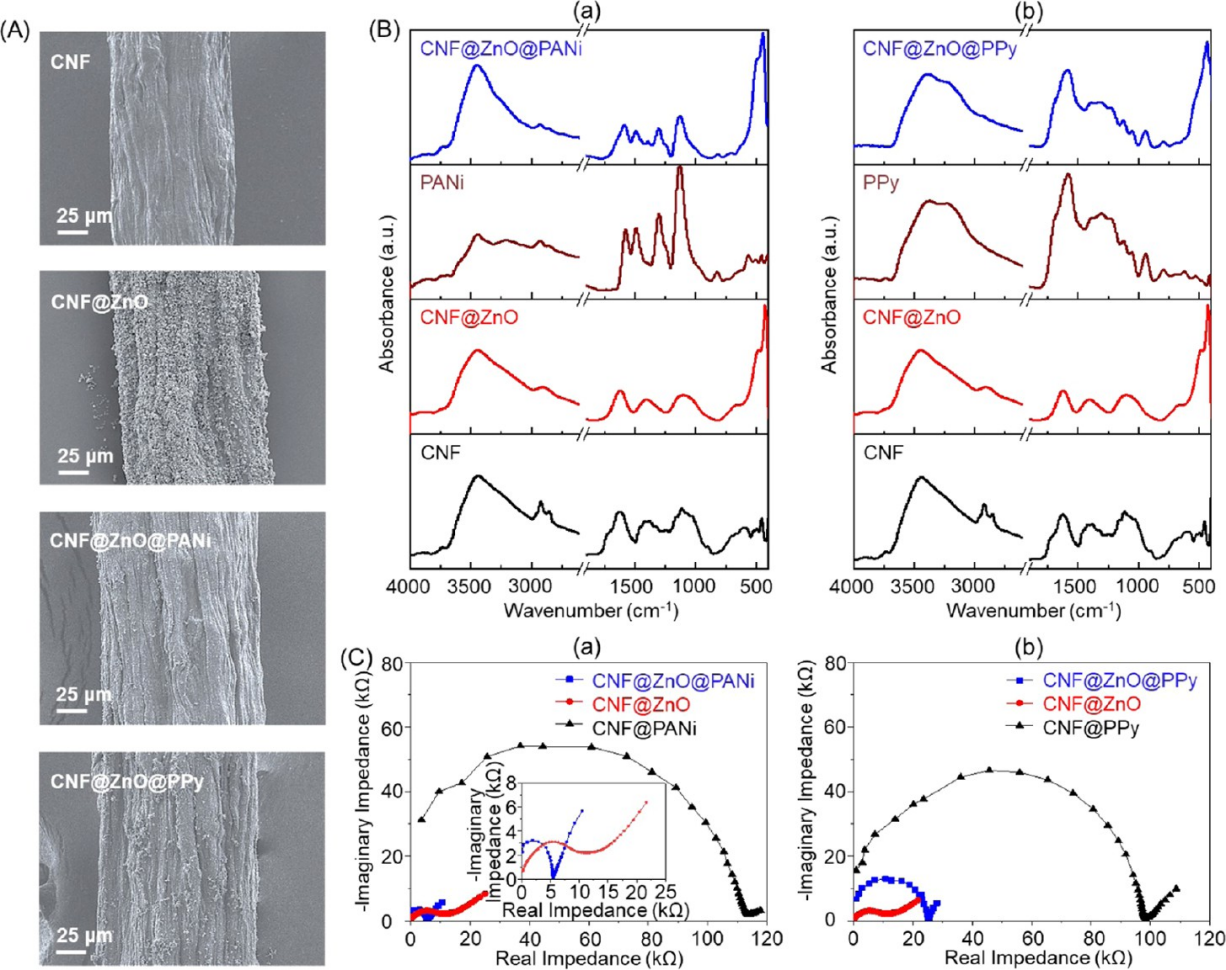
(A) FE-SEM images of CNF, CNF@ZnO, CNF@ZnO@PANi, and CNF@ZnO@PPy filaments. (B) FTIR absorption spectra for (a) CNF@ZnO@PANi and PANi as well as (b) CNF@ZnO@PPy and PPy. The spectra for CNF and CNF@ZnO are included for reference purposes. (C) EIS Nyquist plots of (a) CNF@ZnO@PANi and CNF@PANi filaments and (b) CNF@ZnO@PPy and CNF@PPy filaments. Data for the CNF@ZnO filament are included as a reference.

The two conducting polymers were deposited on the filaments to further examine their electrochemical properties. For this purpose, the filaments carrying ZnO after femtosecond laser deposition were *in situ* polymerized with the aniline or pyrrole monomer. Figure 8A shows the SEM images of CNF@ZnO@PANi and CNF@ZnO@PPy filaments, which were compared with those of the bare CNF and CNF@ZnO filaments. The average diameter (124 μm) of the CNF@ZnO filament increased slightly (to 134 μm) after the polymerization of aniline and pyrrole monomers, suggesting that conductive polymers were polymerized on the surface of the CNF@ZnO filament. This increased diameter (10 μm) is limited compared to the increased diameter (23 and 32 μm, respectively) achieved with the PANI and PPy in CNF@PANi and CNF@PPy, respectively, given the possible penetration of PANI and PPy into the ZnO layer.

The chemical structures of CNF@ZnO@PANi and CNF@ZnO@PPy filaments were compared with those of the CNF@ZnO filament, PANi, and PPy by using FTIR absorption spectroscopy (Figure 8B). The FTIR absorption spectrum of the CNF@ZnO filament was characterized by the bands at 3400, 2899, 1611, 1380, and 1100 cm^–1^ for CNF and 480 nm for ZnO.^27,28^ On the other hand, fingerprint bands of PANi were shown at 1572, 1483, 1300, and 1110 cm^–1^ corresponding to the C=C stretching vibration mode of benzenoid and quinonoid rings, the C–N stretching vibration mode, the C–H bending vibration mode in the benzene ring, and the N–H wagging mode of secondary amine, respectively.^42^ Then, the FTIR spectrum of the CNF@ZnO@PANi filament (Figure 8B(a)) showed mainly similar bands to those of PANi and ZnO, although absorption bands of CNF appeared as a shoulder or weak band. These results suggest the coexistence of CNF, ZnO, and PANi in the hybrid filaments. Major absorption bands of PPy were observed at 3280, 1565, 1410, and 1295 cm^–1^. They are attributed to the N–H, C=C, C–C, and C–N stretching vibration modes.^43^ IR bands of CNF@ZnO@PPy were almost similar to those of PPy besides the addition of a band of ZnO and a shoulder or overlapped bands of CNF, implying the formation of the CNF@ZnO@PPy filament (Figure 8B(b)).

**Figure 9:**
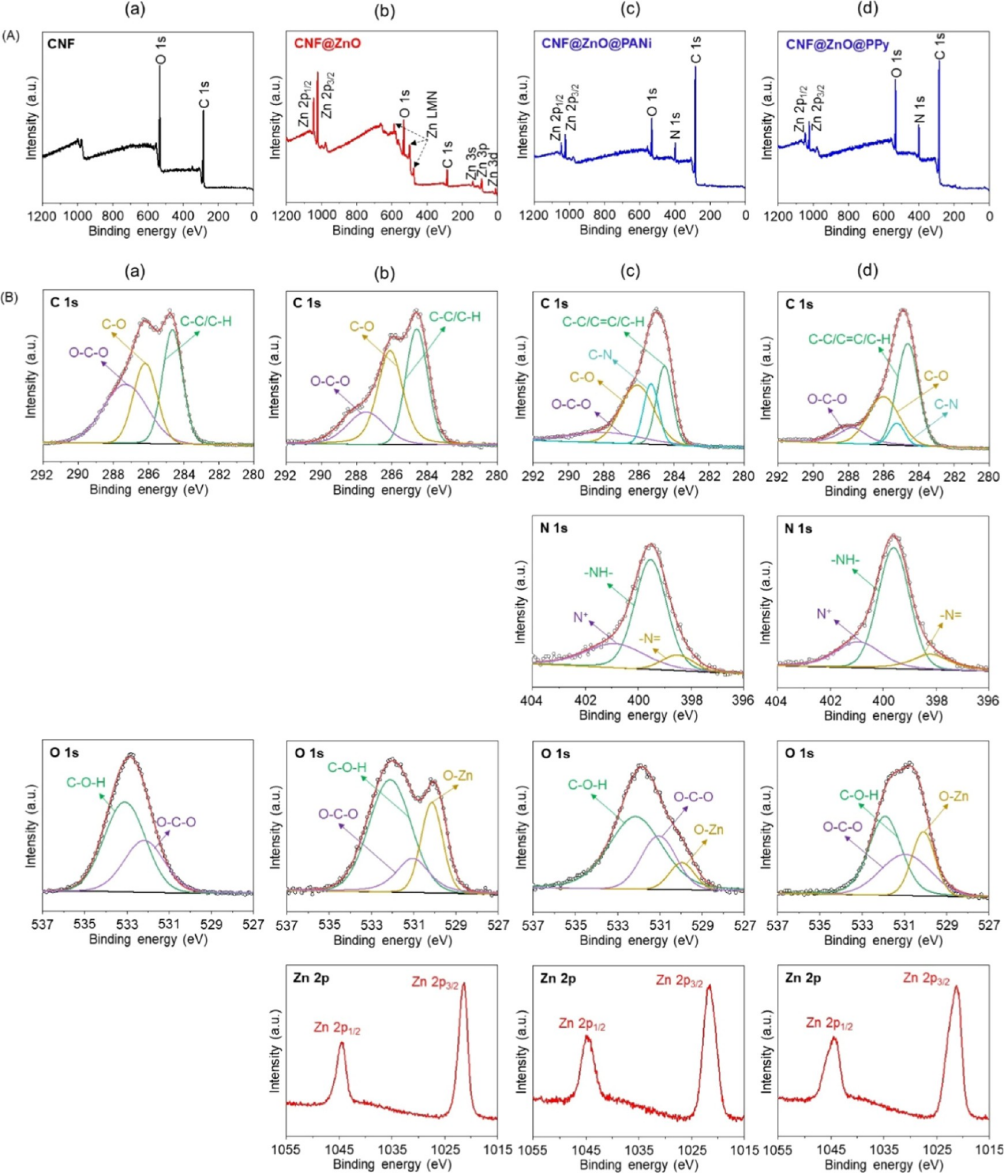
(A) Survey XPS for (a) CNF, (b) CNF@ZnO, (c) CNF@ZnO@PANi, and (d) CNF@ZnO@PPy. Panel (B) includes the fine spectra of C 1s, N 1s, O 1s, and Zn 2p peaks corresponding to CNF (column (a)), CNF@ZnO (column (b)), CNF@ZnO@PANi (column (c)), and CNF@ZnO@PPy (column (d)).

Survey XPS of CNF (Figure 9A(a)), CNF@ZnO (Figure 9A(b)), CNF@ZnO@PANi (Figure 9A(c)), and CNF@ZnO@PPy (Figure 9A(d)) revealed the peaks of the main elements. The fine XPS of each peak was deconvoluted into the corresponding bond peaks. CNF (Figure 9B(a)) exhibited C 1s of O–C–O (287.5 eV), C–OH (286.2 eV), and C–C/C–H (284.6 eV) bonds and O 1s of C–OH (533.1 eV) and O–C–O (532.2 eV) bonds. These bonds appeared even in CNF@ZnO (Figure 9B(b)), CNF@ZnO@PANi (Figure 9B(c)), and CNF@ZnO@PPy (Figure 9B(d)). Meanwhile, C–N (285.3 eV) and C=C (overlapped on the C–C/C–H peak) bonds from PANi and PPy in C 1s were shown in CNF@ZnO@PANi and CNF@ZnO@PPy. The O–Zn (530.0 eV) bond was displayed in CNF@ZnO, CNF@ZnO@PANi, and CNF@ZnO@PPy. Zn 2p_1/2_ (1044.7 eV) and Zn 2p_3/2_ (1021.5 eV) bonds were invariant among CNF@ZnO, CNF@ZnO@PANi, and CNF@ZnO@PPy. The N 1s peaks corresponding to N^+^ (401.1 or 409.9 eV), –NH– (399.5 or 399.6 eV), and –N= (398.4 or 398.2 eV) were apparent in CNF@ZnO@PANi and CNF@ZnO@PPy, respectively.^44,45^ The results of XPS confirm the successful synthesis of CNF composites.

Figure 8C(a) shows the EIS Nyquist plots of the CNF@PANi, CNF@ZnO, and CNF@ZnO@PANi filament electrodes in 1 M NaCl. Their respective values of charge transfer resistance were 121, 11, and 5.5 kΩ corresponding to 0.21, 2.33, and 4.31 mS/cm in conductivity. The results indicate that the electron transfer in the CNF@ZnO@PANi filament electrode is up to 20 and 2 times faster than those for CNF@PANi and CNF@ZnO filament electrodes, respectively. EIS data confirm that the CNF@ZnO@PANi filament processes a significantly enhanced conductivity due to the synergistic contribution of ZnO and PANi. Figure 8C(b) displays the EIS Nyquist plots of CNF@PPy, CNF@ZnO, and CNF@ZnO@PPy filament electrodes. All filament electrodes showed semicircles, indicating good conductive characteristics, and the CNF@PPy filament revealed a maximum radius at 99 kΩ corresponding to a conductivity of 0.24 mS/cm, while CNF@ZnO@PPy approached a smaller resistance value at 25.6 kΩ corresponding to a conductivity of 0.93 mS/cm. However, a CNF@ZnO@PPy filament displayed lower conductivity than the CNF@ZnO filament, implying that the conductivity of a CNF@ZnO@PPy electrode followed an additive rule as far as the conductive ZnO and PPy. This phenomenon was drastically different from the result of the conductivity of the CNF@ZnO@PANi system, which indicated a synergistic effect of conductive ZnO and PANi.

**Figure 10:**
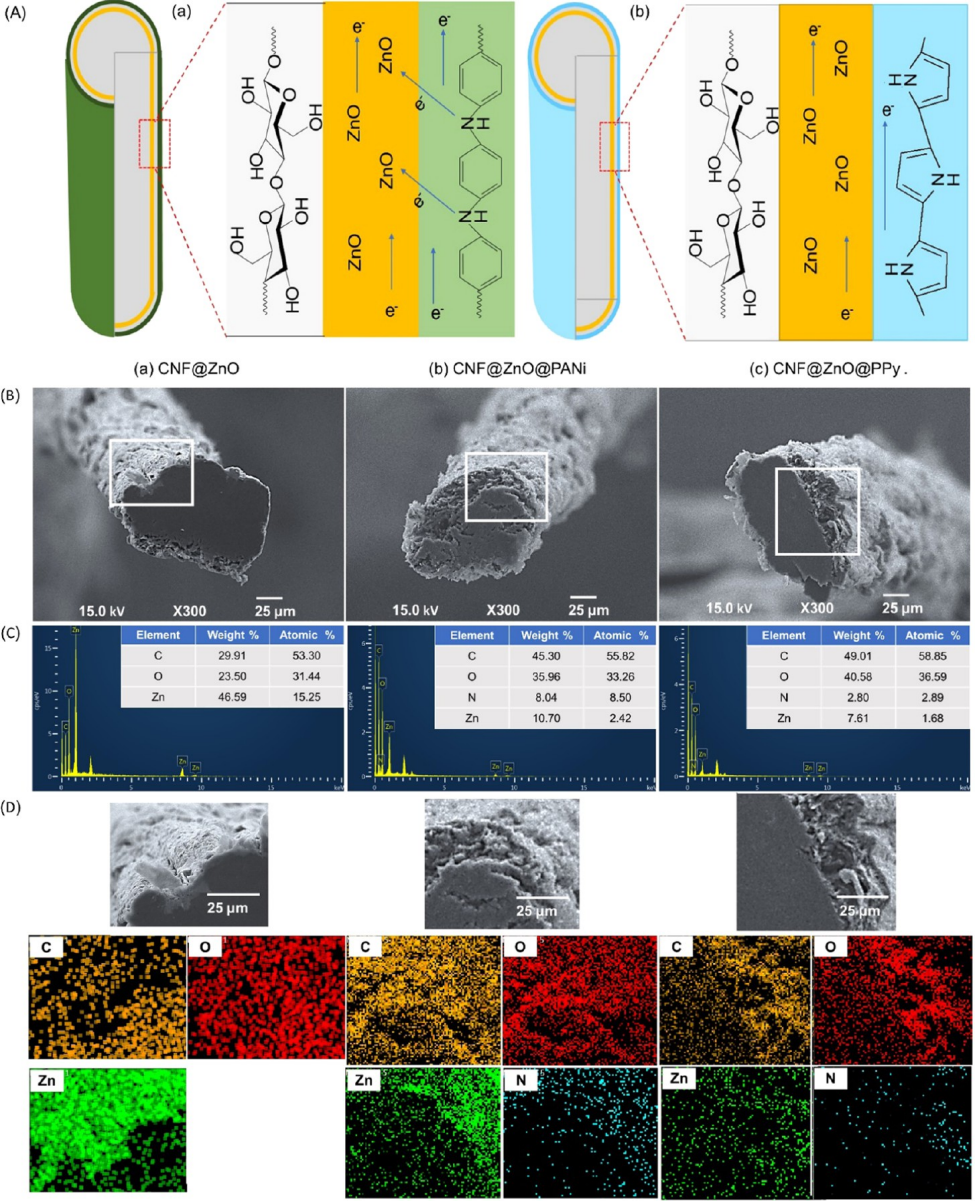
(A) Schematic illustration of double-layer structures in (a) CNF@ZnO@PANi and (b) CNF@ZnO@PPy filaments. (B) FE-SEM images and (C) EDX of filaments and sections and (D) elemental mapping of carbon, oxygen, nitrogen, and zinc corresponding to (a) CNF@ZnO, (b) CNF@ZnO@PANi, and (c) CNF@ZnO@PPy.

A possible reason for the difference in conductivity of the two filament electrodes may be related to the energy level alignment of the electrodes. Since the conductive band energy of ZnO is lower than the LUMO of PANI and PPy, electrons of the conductive polymer can transfer to ZnO, but since the LUMO of PPy is lower than that of PANI, electron transfer at ZnO@PPy must be faster than ZnO@PANi (see Figure S6). Unfortunately, this hypothesis disagrees with the observed results. An alternative explanation relates to the electron transfer between ZnO and PC (Figure 10A). Since the lone pair of an amine group in PANi can work as an electron donor and ZnO acts as an acceptor, electron transfer increases. On the other hand, lone pair electrons of amine in the heterocyclic pyrrole ring are delocalized and, thus, electrons independently transport in PPy and ZnO layers. Different effects were observed on the supercapacitor performance of PANi-carbon dot- and PPy-carbon dot-based electrodes, too: The former capacitance was synergetic, but the latter was additive.^42^

Figure 10B shows the FE-SEM images of filaments and their cross-sections of the filaments. The CNF filaments exhibited densely packed features and flat surfaces (Figure S7). For CNF@ZnO, CNF@ZnO@PANi, and CNF@ZnO@PPy filaments, which were prepared under optimal conditions, EDX analyses indicated the coexistence of carbon, oxygen, and zinc elements in the CNF@ZnO filament and an additional nitrogen element in CNF@ZnO@PANi, and CNF@ZnO@PPy filaments (Figure 10C).

It should be noticed that the sectional density in CNF@ZnO was homogeneous, but after loading with PANi or PPy, the periphery in the cross-section was rather rough or less dense. Moreover, elemental mapping profiles (Figure 10D) indicate that zinc and nitrogen elements were evenly distributed in the whole cross-section. Hence, it may be anticipated that precursor Zn ions penetrated in the CNF filament and were oxidized into ZnO; similarly, the PANi and PPy monomers (aniline or pyrrole, respectively) penetrated partially in CNF@ZnO and were polymerized *in situ*. Thus, layer boundaries were further fused by the partial penetration of loaded ZnO in CNF and polymers in CNF@ZnO. A similar penetration of loaded PANi in CNF is apparent in Figure S8.

A comparison with other conductive filaments is provided in Table 2, which includes hierarchical structures made from PANi incorporated in cellulose nanocrystals/natural rubber^18^ or CNF aerogels,^19^ all of which showed a relatively low conductivity. By contrast, a high conductivity was measured for membranes composed of PANi and bacterial cellulose^46,47^ and for CNF films.^48^ Films based on chitosan and PPy showed relatively low conductivity,^49^ while that of cellulose hydrogels was relatively high.^50^ Our filaments displayed a low conductivity compared to PANi or PPy in two- and three-dimensional insulators. Although there are no previous reports on double conductive materials, PANi/ZnO nanocomposites have been reported to have a higher conductivity than PPy/ZnO nanocomposites.^51−53^ Overall, the performance of our CNF filaments based on PANi/ZnO is comparable to or better than that of the corresponding nanocomposites.

**Table 2:** Conductivity Values are in Comparison with Previous Related Studies

material	conductivity (mS/cm)	reference
cellulose nanocrystals/natural rubber/PANi 3D hierarchical structure	0.54	(18)
cellulose nanofibril/PANi aerogel	0.37	(19)
bacterial cellulose/PANi membrane	1.04	(46)
bacterial cellulose/PANi membrane	4.5	(47)
cellulose nanofibril/PANi film	10	(48)
chitosan/PPy film	10^–4^–10^0^	(49)
cellulose/PPy hydrogel	9.18	(50)
PPy/ZnO nanocomposite	1.42 × 10^–4^	(51)
PANi/ZnO nanocomposite	4.35	(52)
PANi/ZnO nanocomposite	1.4–6.8	(53)
CNF/ZnO hybrid filament	2.33	this work
CNF/PPy hybrid filament	0.24	this work
CNF/PANi hybrid filament	0.21	this work
CNF/ZnO/PPy hybrid filament	0.93	this work
CNF/ZnO/PANi hybrid filament	4.31	this work

## 4 Conclusions

Conductive filament electrodes based on nanocellulose (CNF@ZnO, CNF@PANi, CNF@PPy, CNF@ZnO@PANi, and CNF@ZnO@PPy) were successfully synthesized by the deposition of a conductive metal oxide along with a conductive polymer (PANI or PPy). The conductivity of the CNF@ZnO filament prepared via femtosecond laser irradiation was nearly 6-fold higher than the one obtained through deposition by the polyol solvent method. This is explained by the coating on the surface of nanoparticles produced by the latter method (bare ZnO nanoparticles carry no protector or stabilizer, which is preferable for electron transport). CNF@PANi and CNF@PPy synthesized via *in situ* polymerization were conductive (though their conductivity was inferior to that of CNF@ZnO filaments modified by femtosecond pulse laser deposition). In addition, the electrical conductivity of CNF@PANi and CNF@PPy filaments depended on the polymer loading: excess polymer led to diameter and conductivity that tended to progressively diminish. The amount of APS (oxidizing agent used in the polymerization) relative to the monomer and the process used for APS addition affected the polymerization and conductivity.

The filaments carrying a double layer (CNF@ZnO@PANi and CNF@ZnO@PPy) showed outstanding conductivity through the cooperative effect of the metal oxide and the conducting polymer: the coexistence of PANi and ZnO on the CNF filament synergistically enhanced the conductivity due to electron transfer from amine (PANi) to ZnO; meanwhile, the conductivity of CNF@ZnO@PPy filaments was additive (PPy and ZnO). Overall, thin (ca. 100 μm) and highly conductive filaments were prepared by combining a ceramic material and a sustainable polymeric structure. These new hybrid filaments compete with inorganic counterparts made from silicon and germanium and are valuable for applications that negate the use of metals. The hybridization of graphene oxide in CNF filaments was performed and the enhancement of mechanical strength was confirmed for hybrid filaments,^54^ although their mechanical strength was still lower than that of modified CNF filaments.^55^ Thus, the development of hybrid filaments with strong mechanical strength is expected in future research.

## Supporting Information Available

FE-SEM images, FTIR absorption spectra, and Nyquist plots of CNF@ZnO filaments; optical FE-SEM images, diameter and PPy weight variations, Nyquist plots, and conductivity variations of CNF@PPy filaments; and energy level alignments of ZnO@PANi and ZnO@PPy (PDF)
