# Supplementary material for: Nanoarchitectonics of Nanocellulose Filament Electrodes by Femtosecond Pulse Laser Deposition of ZnO and In Situ Conjugation of Conductive Polymers
Source: ACS Appl Mater Interfaces. 2024 Apr 17;16(17):22532–46. doi: 10.1021/acsami.4c02780 (PMC11071050; doi:10.1021/acsami.4c02780)
Supplement: Supplementary file 1 — am4c02780_si_001.pdf [file am4c02780_si_001.pdf]

Supporting Information

Nanoarchitectonics of nanocellulose filament electrodes by femtosecond pulse laser deposition of ZnO and *in-situ* conjugation of conductive polymers

Duong Tuan Anh Nguyen,[1] *Ling Wang,*[2] *Toyoko Imae,*[1,3,*] *Chun-Jen Su,*[4] *U-Ser Jeng,*[4,5] *Orlando J. Rojas*[2,6,*]

[1] Graduate Institute of Applied Science and Technology, National Taiwan University of Science and Technology, Taipei 10607, Taiwan

[2] Department of Bioproducts and Biosystems, School of Chemical Engineering, Aalto University, 00076, Finland

[3] Department of Chemical Engineering, National Taiwan University of Science and Technology, Taipei 10607, Taiwan

[4] National Synchrotron Radiation Research Center, Hsinchu, 300092, Taiwan

[5] Department of Chemical Engineering & College of Semiconductor Research, National Tsing Hua University, Hsinchu 300044, Taiwan

[6] Bioproducts Institute, Department of Chemical and Biological Engineering, Department of Chemistry and Department of Wood Science, University of British Columbia, Vancouver, V6T 1Z3, BC, Canada

*Corresponding authors e-mails: imae@mail.ntust.edu.tw (T.I.), orlando.rojas@ubc.ca (O.J.R.)

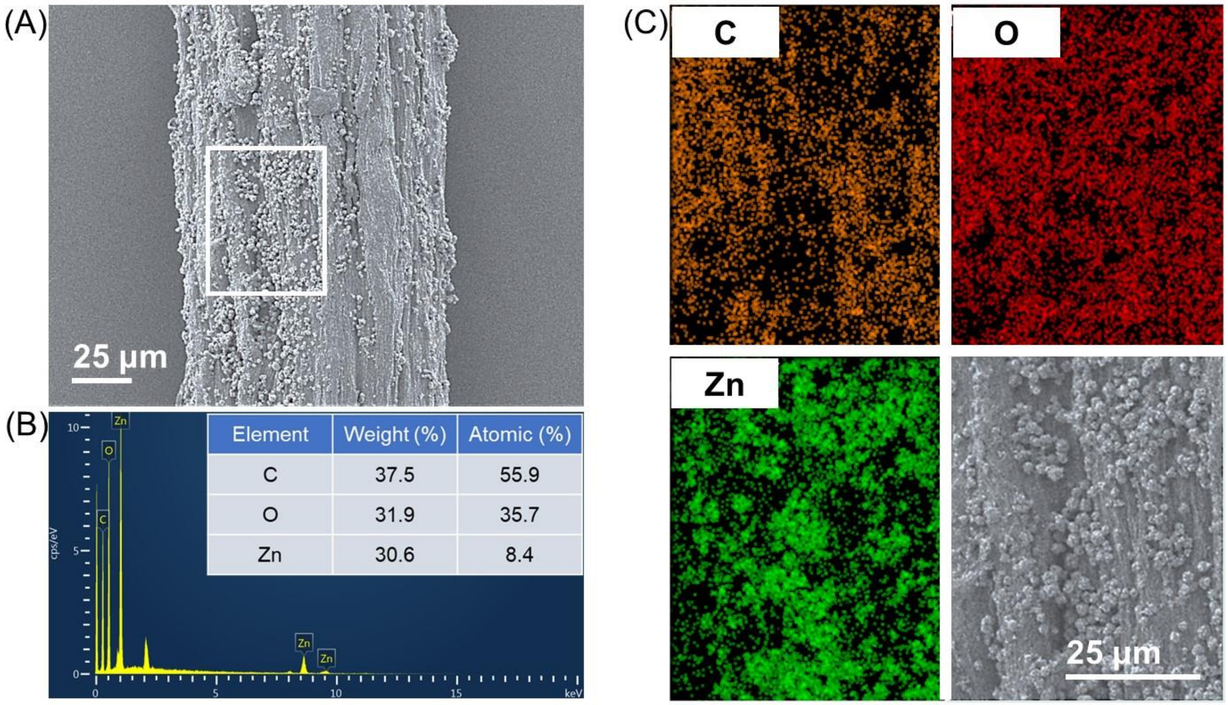

Figure S1. (A) FE-SEM image, (B) EDX, and (C) elemental mapping of carbon, oxygen, and zinc in a rectangular image of CNF@ZnO filament prepared by femtosecond laser irradiation deposition at 0.1 M precursor concentration and 1 h holding time.

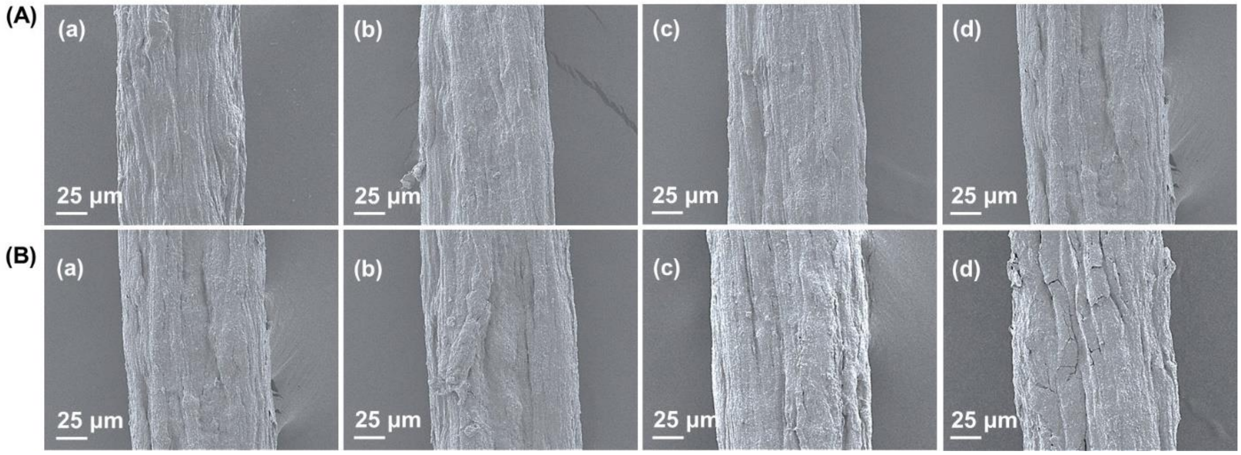

Figure S2. FE-SEM images of CNF@ZnO filaments prepared in polyol solvent (A) at different adsorption periods of precursor Zn ion of a) 0, (b) 1, (c) 10, and (d) 20 h for 1 h incubation, and (B) at different incubation periods of (a) 1, (b) 5, (c) 10, and (d) 20 h for 20 h adsorption of Zn ion.

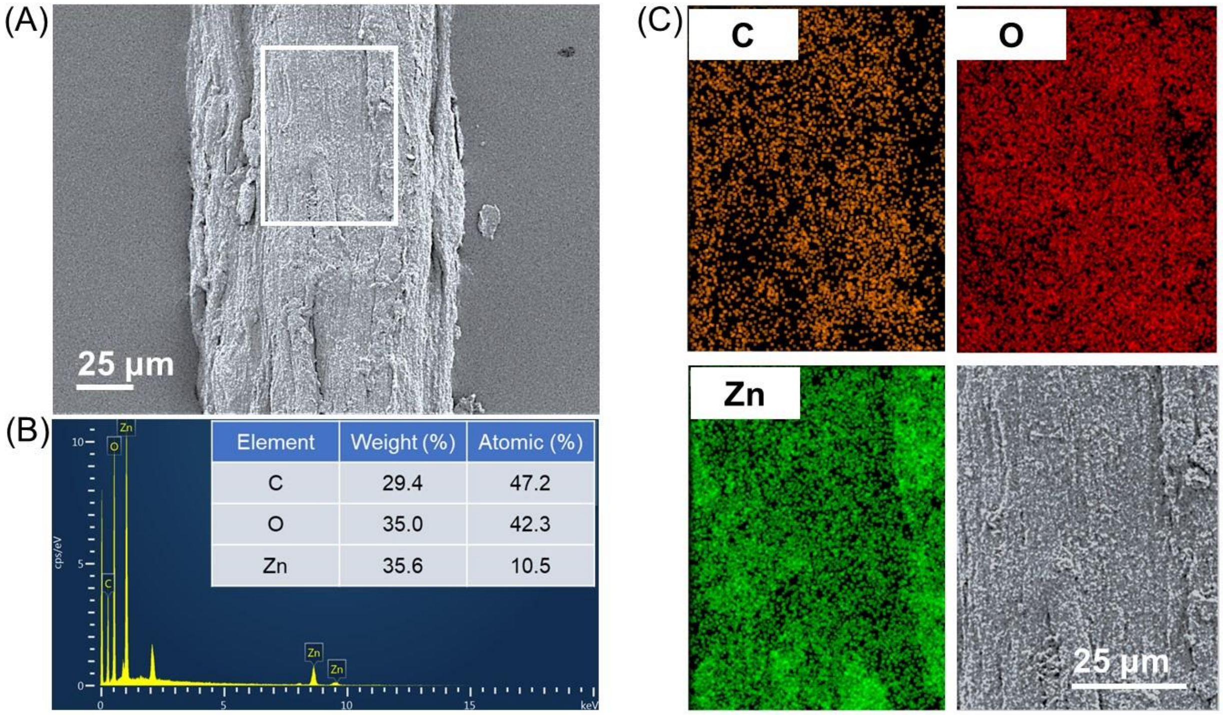

Figure S3. (A) FE-SEM image, (B) EDX, and (C) elemental mapping of carbon, oxygen, and zinc in a rectangular image of CNF@ZnO filament prepared in polyol solvent at 20 h adsorption period of precursor Zn ion and 1 h incubation.

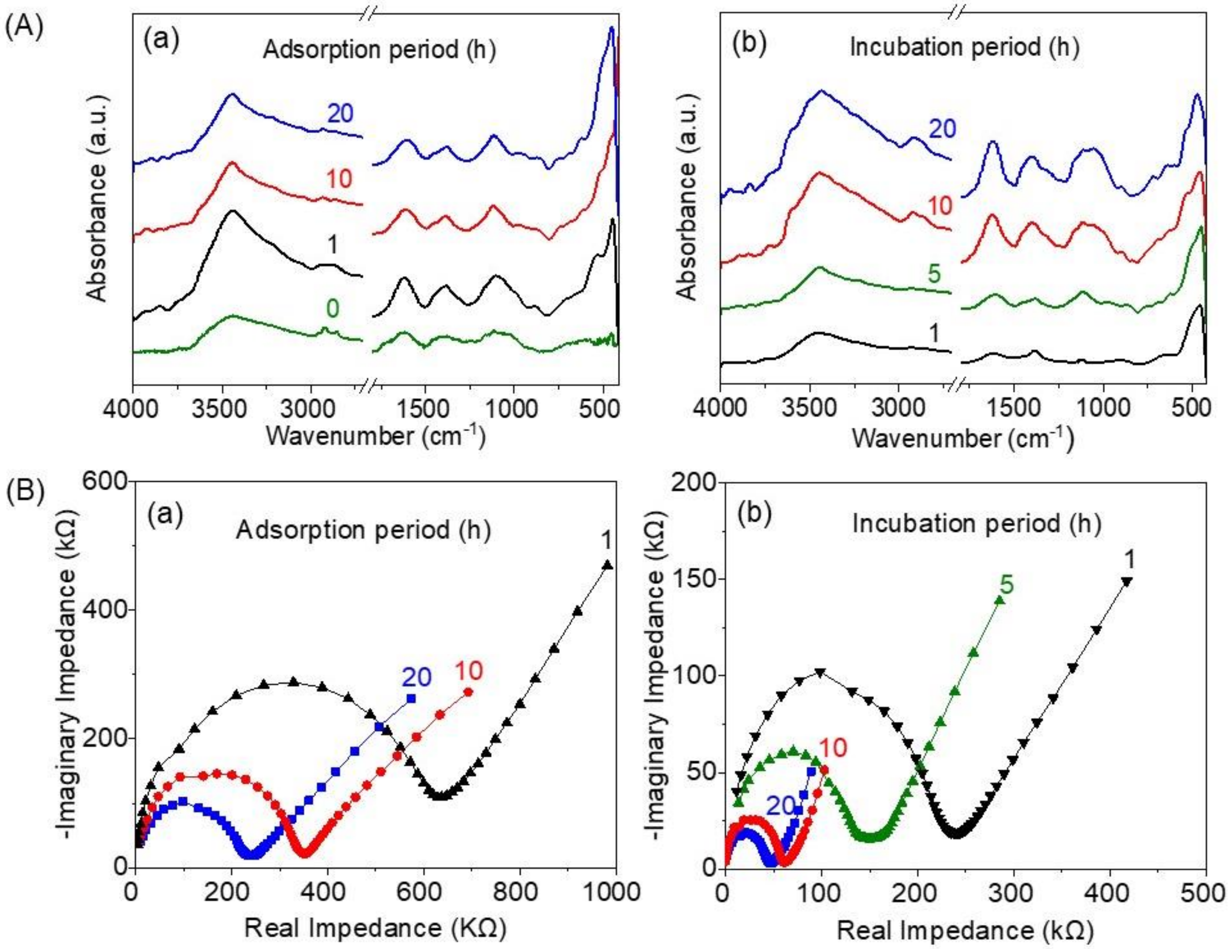

Figure S4. (A) FTIR absorption spectra and (B) EIS Nyquist plots of CNF@ZnO filaments prepared in polyol solvent. (a) at different adsorption periods of 0, 1, 10, and 20 h and (b) at different incubation periods of 1, 5, 10, and 20 h.

All FTIR spectra in both Figure 3A and Figure S2A showed characteristic absorption bands of O-H and C-H stretching vibration modes at 3400 and 2899 cm^{-1}, respectively. Bands at 1611, 1380 and 1100 cm^{-1} were assigned to vibration modes of O-H bending, C-H bending + C-O stretching, and C-O-C bending in polysaccharide rings [1]. The characteristic band of the composite at 480 cm^{-1} corresponds to the Zn-O stretching mode and it was intensified with the increase of adsorption periods, indicating the increase of adsorbed ZnO with time [2].

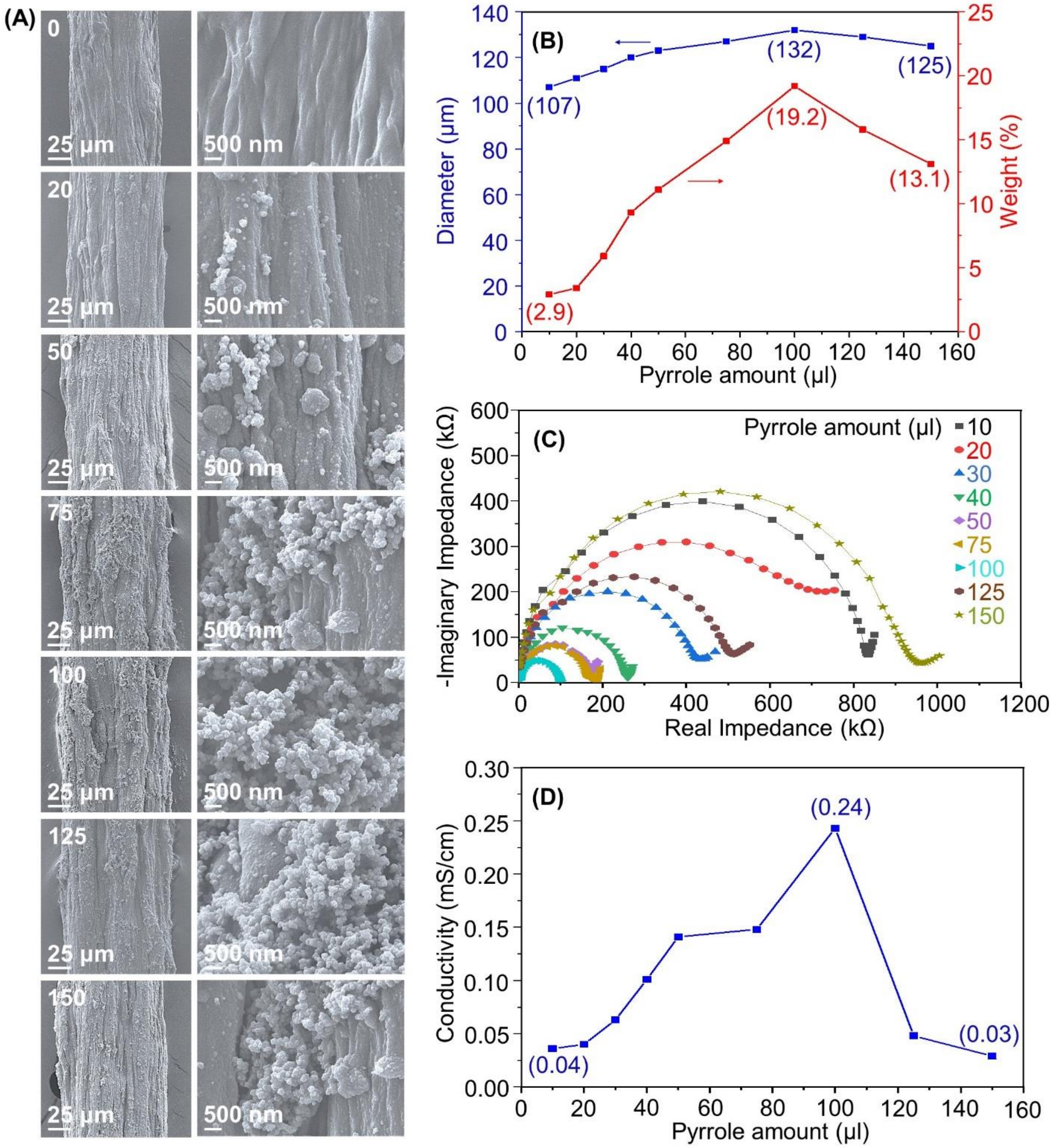

Figure S5. (A) FE-SEM and its magnified images, (B) diameter and PPy weight variations, (C) Nyquist plots, and (D) conductivity variations of CNF@PPy filaments prepared at different amounts of pyrrole monomer.

Table S1. Parameters from EIS curves of CNF@PANi and CNF@PPy filament electrodes prepared at different PANI and PPy loadings.

Filament	Condition		Thickness, µm	Charge transfer resistance, kΩ	Conductivity, mS/cm
CNF@PANi	APS adsorbed on CNF after monomer addition	10 µl	105	356	0.08
		20	111	338	0.09
		30	115	250	0.11
		40	118	185	0.15
		50	120	169	0.16
		75	123	121	0.21
		100	118	136	0.20
		125	109	405	0.07
		150	108	506	0.06
	APS adsorbed on CNF before monomer addition	10 µl	106	945	0.03
		20	108	750	0.04
		30	112	670	0.04
		40	114	541	0.05
		50	116	442	0.06
		75	120	293	0.09
		100	123	218	0.12
		125	128	179	0.14
		150	131	132	0.18
CNF@PPy	APS adsorbed on CNF after monomer addition	10 µl	107	835	0.04
		20	111	723	0.04
		30	115	436	0.06
		40	120	261	0.10
		50	123	183	0.14
		75	127	169	0.15
		100	132	99	0.24
		125	129	513	0.05
		150	125	953	0.03

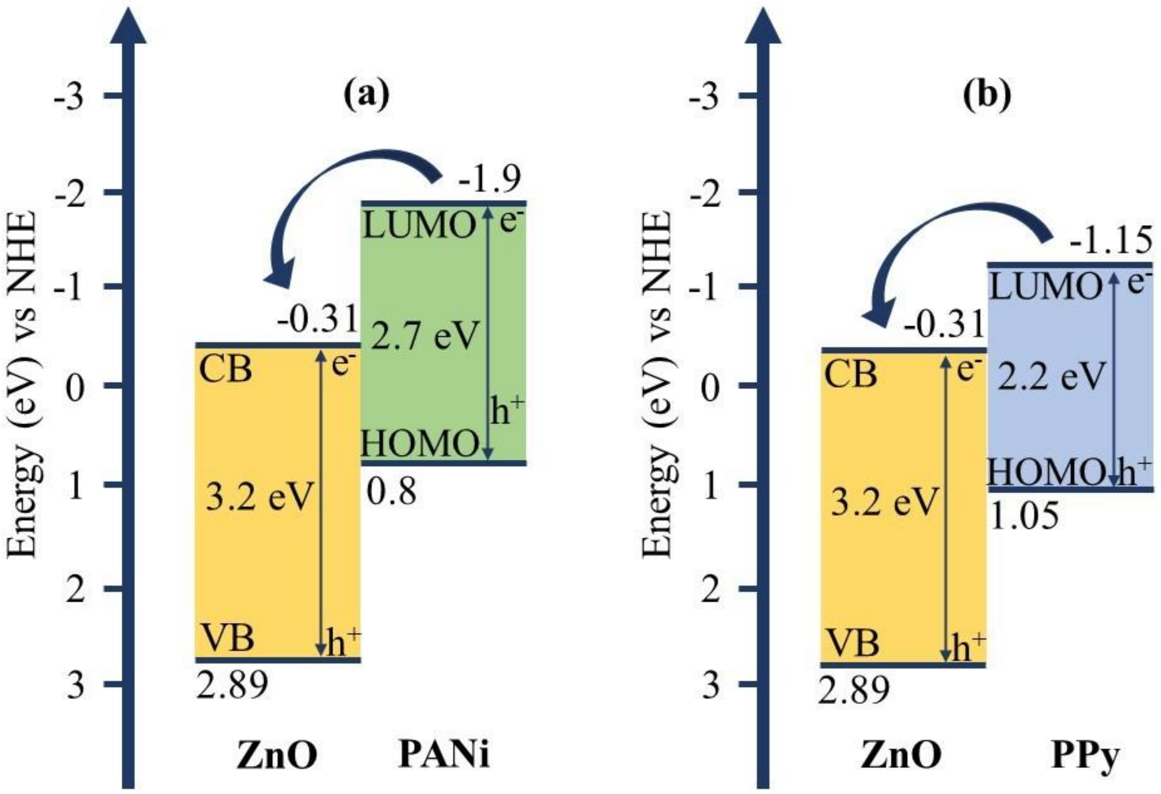

Figure S6. Energy level alignments of (a) ZnO@PANi and (b) ZnO@PPy. Energy levels of ZiO and LUMO and HOMO levels of PANi and PPy were adopted values from literatures [3-5].

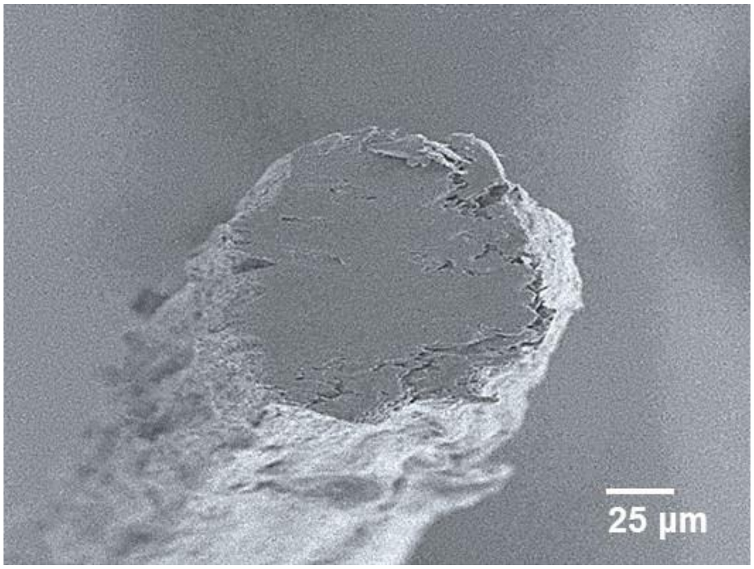

Figure S7. An FE-SEM image of filament and section of CNF.

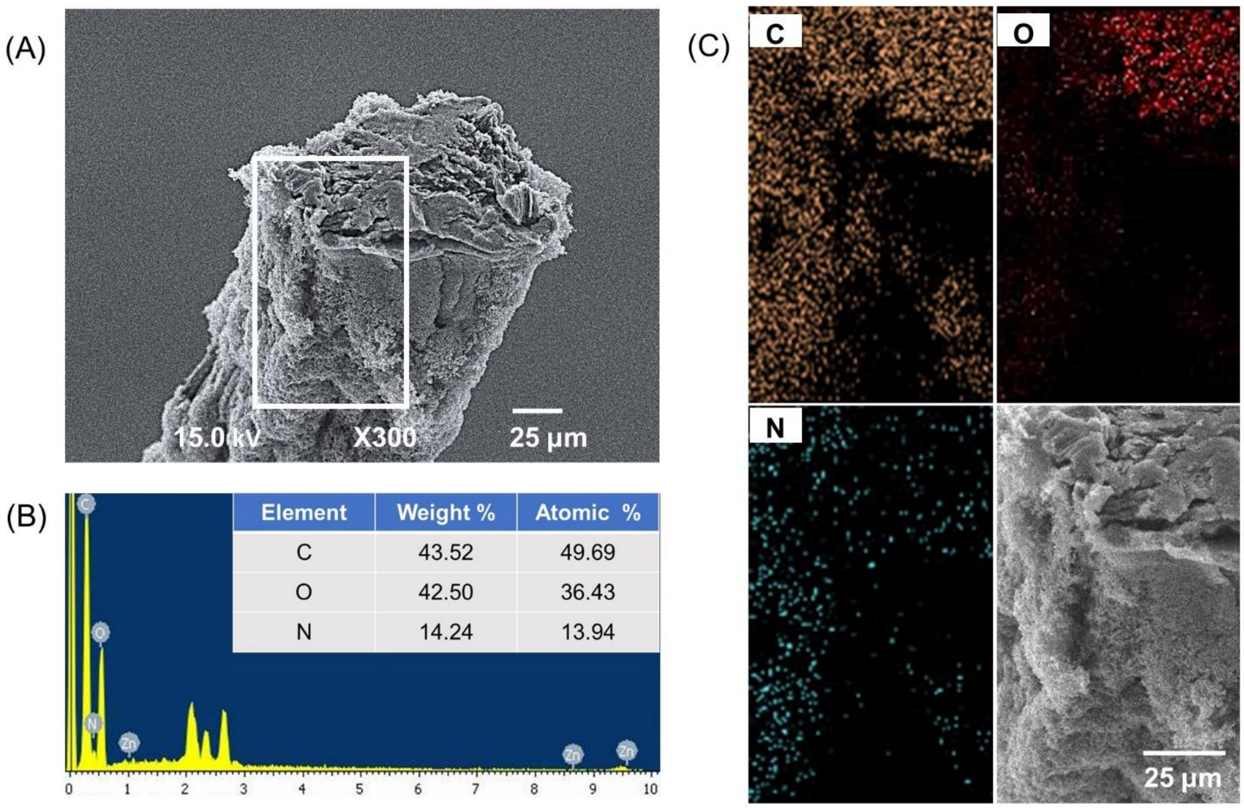

Element	Weight %	Atomic %
C	43.52	49.69
O	42.50	36.43
N	14.24	13.94

Figure S8. (A) FE-SEM images of filaments and sections, (B) EDX of filaments and sections, and (C) elemental mapping of carbon, oxygen, and nitrogen in a rectangular image of CNF@PANi (diameter: 127±2 μm).

References

[1] T.T.A. Do, S. Grijalvo, T. Imae, M.J. Garcia-Celma, C. Rodríguez-Abreu, A nanocellulose-based platform towards targeted chemo-photodynamic/photothermal cancer therapy, *Carbohydr. Polym.* 270 (2021) 118366.

[2] T.A. Geleta and T. Imae, Nanocomposite Photoanodes Consisting of p-NiO/n-ZnO Heterojunction and Carbon Quantum Dot Additive for DyeSensitized Solar Cells, *ACS Appl. Nano Mater.* 2021, 4, 236−249.

[3] G.F. Gemeda, W.-J. Hwang, T. Imae, Y.-W. Yen, J. Colloid Interface Sci. 614 (2022) 310–321.

[4] Q. Wang, J. Hui, J. Li, Y. Cai, S. Yin, F. Wang, B. Su, Appl. Surface Sci. 283 (2013) 577–583.

[5] J. Zia, J. Kashyap, U. Riaz, J. Molecular Liquids 272 (2018) 834–850.
